# Preliminary Characterization of Two Small Insulinase-Like Proteases in *Cryptosporidium parvum*

**DOI:** 10.3389/fmicb.2021.651512

**Published:** 2021-05-21

**Authors:** Rui Xu, Cong Lai, Fuxian Yang, Qiang Zhang, Na Li, Yaqiong Guo, Lihua Xiao, Yaoyu Feng

**Affiliations:** ^1^State Key Laboratory of Bioreactor Engineering, School of Resources and Environmental Engineering, East China University of Science and Technology, Shanghai, China; ^2^Center for Emerging and Zoonotic Diseases, College of Veterinary Medicine, South China Agricultural University, Guangzhou, China

**Keywords:** *Cryptosporidium parvum*, insulinase-like protease, localization, invasion, expression

## Abstract

*Cryptosporidium parvum* is a major cause of moderate-to-severe diarrhea in humans and animals. Its compact genome contains 22 genes encoding divergent insulinase-like proteases (INS), which are poorly characterized. In this study, two small members of this family, INS-21 encoded by cgd7_2080 and INS-23 encoded by cgd5_3400, were cloned, expressed, and characterized to understand their functions. Recombinant INS-21 and INS-23 were expressed in *Escherichia coli* and polyclonal antibodies against these two proteins were prepared. The cgd7_2080 gene had a high transcription level during 0–2 h of *in vitro C. parvum* culture, while cgd5_3400 was highly transcribed at 0–6 h. INS-21 was mostly located in the apical region of sporozoites and merozoites whereas INS-23 was found as spots in sporozoites and merozoites. The immunoelectron microscopy confirmed the expression of INS-21 in the apical region of sporozoites while INS-23 appeared to be expressed in the dense granules of sporozoites. The neutralization efficiency was approximately 35%, when the cultures were treated with anti-INS23 antibodies. These results suggest that INS-21 and INS-23 are expressed in different organelles and might have different functions in the development of *C. parvum*.

## Introduction

*Cryptosporidium* spp. are common protozoan pathogens, causing the enteric disease cryptosporidiosis in humans and animals at early age (Kotloff, 2017). Over 40 species have been recognized, but most human cases of cryptosporidiosis are caused by *C. parvum* and *C. hominis* (Feng et al., 2018). Currently, approximately 8.6% of global child mortality is caused by diarrheal diseases (Liu L. et al., 2016), with *Cryptosporidium* as one of the top five pathogens for moderate-to-severe diarrhea in children under 5 years in developing countries (Khalil et al., 2018; Platts-Mills et al., 2018; Kotloff et al., 2019). Nitazoxanide is the only drug approved by FDA for the treatment of cryptosporidiosis. However, it has only limited efficacy in infants and immunocompromised individuals who have the most urgent need of treatment (Bhalchandra et al., 2018).

Poor understanding of the biology of *Cryptosporidium* spp. is one of the main obstacles in the development of effective drugs and vaccines against cryptosporidiosis. *Cryptosporidium* spp. have a complex life cycle including excystation, invasion, merogony, gametogony, oocyst formation, and sporulation (Bouzid et al., 2013). In contrast to most other apicomplexans, the entire life cycle of *Cryptosporidium* spp. occurs in a single host. After the host has ingested oocysts, sporozoites are released from them to invade the intestinal epithelial cells. The parasites undergo multiple rounds of asexual replication before 36 h post-infection (hpi) and enter the sexual phase forming macrogamonts or microgamonts at 44–48 hpi (Tandel et al., 2019; Funkhouser-Jones et al., 2020).

Metallopeptidases have diverse functions in parasitic protozoa and play crucial roles in their biology (Escotte-Binet et al., 2018). The M16 family of zinc-metallopeptidases are characterized by the presence of an inverted zinc binding motif “HXXEH” (X could be any amino acid) and have three subfamilies (M16A, M16B, and M16C) based on their primary structure. M16 metalloproteases have diverse roles in cells and can be found in cytosol, peroxisome, endosome, and the cell surface (Rawlings et al., 2014). M16 metalloproteases are also common in apicomplexans, but *Cryptosporidium* spp. appear to have more members than other apicomplexans. Two M16 metallopeptidases have been described in *Toxoplasma gondii*, both belonging to the M16A subfamily. They are found in rhoptries and micronemes, which play key roles in host cell interactions (Laliberte and Carruthers, 2011; Hajagos et al., 2012). In contrast, the two M16 metallopeptidases identified in *Plasmodium falciparum* belong to the M16C subfamily. They were shown to participate in hemoglobin degradation and the transit peptides of apicoplast protein degradation (Eggleson et al., 1999; van Dooren et al., 2002).

The *C. parvum* genome contains 22 genes encoding M16 metallopeptidases (Liu S. et al., 2016), 18 of which belong to the M16A subfamily, 3 to the M16B subfamily, and 1 to the M16C subfamily. They have divergent sequences and contain one to four M16 domains. Thus far, only three of them have been characterized biologically, including INS-5, INS-15, and INS-20-19 (Xu et al., 2019; Zhang et al., 2019; Ni et al., 2020). In this study, we have focused on two small M16B proteases of *C. parvum*: INS-21 encoded by the cgd7_2080 gene and INS-23 encoded by the cgd5_3400 gene. They both have two M16 domains; INS-23 has one active domain with an active site “HFLEH” followed with one inactive domain, while INS-21 has two inactive domains. We conducted some preliminary characterization of INS-21 and INS-23, exploring their roles in invasion and parasites growth.

## Materials and Methods

All experiments and processes in this study adhere to biosecurity and institutional safety procedures.

### Parasites and Host Cells

*Cryptosporidium parvum* oocysts (IOWA isolate) were purchased from Waterborne, Inc. (New Orleans, LA, United States) and stored in PBS with penicillin, streptomycin, amphotericin B, and 0.01% Tween 20 at 4°C for less than 2 months. Prior to infection, oocysts were treated with 0.5% sodium hypochlorite on ice for 10 min and washed three times with PBS buffer. To obtain sporozoites, hypochlorite-treated oocysts were suspended in PBS buffer containing 0.75% taurodeoxycholic acid and 0.25% trypsin and incubated at 37°C for 1 h. The sporozoites released were harvested by centrifuging at 1300 *g* for 3 min. Genomic DNA was extracted from 6 × 10^6^ *C. parvum* oocysts using the Qiagen DNeasy Blood & Tissue Kit (Qiagen, Hilden, Germany) and stored at −20°C for future use.

Human ileocecal adenocarcinoma HCT-8 cells were obtained from the Shanghai Branch of the Chinese Academy of Sciences. They were seeded into 12-well cell culture plates and cultured in RPMI 1640 medium containing 10% fetal bovine serum (FBS), 100 units/ml penicillin, and 100 μg/ml streptomycin at 37°C until ∼90% confluence. Each well was infected with 5 × 10^5^ hypochlorite-treated oocysts in 2% FBS-supplemented RPMI 1640 medium. After 2-h incubation, uninvaded parasites were washed off the culture with PBS. Fresh 2% FBS-supplemented RPMI 1640 medium was added to the culture, which was maintained for specified duration depending on the assay.

### Cloning, Expression, and Purification of INS Proteins

Sequences of the INS-21 (gene name: cgd7_2080) and INS-23 (gene name: cgd5_3400) genes were retrieved from the CryptoDB^1^ and function domains in them were predicted using Pfam 31.0^2^. Sequence alignment of INS-21, INS-23, and the α and β units of mitochondrial processing peptidase (MPP) from *Starmerella bombicola* (yeast) were made using ClustalX^3^. The cgd7_2080 gene was amplified using primers 5′-CCCGAGCTCATGATTCAACAGAAAAT-3′ (the *Sac*I restriction site underlined) and 5′-CCC AAGCTTATAATTGCTGACTTG-3′ (the *Hin*dIII restriction site underlined), while the cgd5_3400 gene was amplified using primers 5′-CGGGATTCTCCATACTAAAACTTGGATG (the *Bam*HI restriction site underlined) and 5′-CCG CTCGAGAGAATAGGAATGAAGATA (the *Xho*I restriction site underlined). The PCR was performed in a GeneAmp 9700 (Applied Biosystems, Foster City, CA, United States) with the following cycling conditions: 95°C for 5 min; 35 cycles of 95°C for 45 s, 55°C for 45 s, 72°C for 2 min; and 72°C for 7 min. The PCR products were purified and cloned into pET-28a (Novagen, Madison, WI, United States). The recombinant plasmids generated were transformed into *Escherichia coli* DH5α competent cells, with positive clones being identified by PCR using the T7/T7t universal primers. The sequence accuracy of the cloned genes was confirmed by DNA sequencing. The plasmids were named pET-28a-INS21 or pET-28a-INS23.

For the expression of recombinant INS-21 and INS-23, the pET-28a-INS21 or pET-28a-INS23 plasmid was transformed into *E. coli* BL21 (DE3) cells. For the mini culture, the single colony of each transformant was cultured at 37°C and 200 rpm overnight in 5 ml of LB medium containing 100 μg/ml kanamycin. The cells from the culture were transferred into 500 ml of LB medium containing 100 μg/ml kanamycin, which was cultured at 37°C and 200 rpm until the OD_600_ reaches 0.6–0.8. At this time point, isopropyl β-D-1-thiogalactopyranoside (IPTG) at a final concentration of 0.5 mM was introduced to induce protein expression, with the culture being maintained at 18°C and 200 rpm for 8 h. The cells were harvested by centrifuging at 4000 *g* for 10 min and stored in −20°C for future use. The expression level of the target protein was evaluated using the SDS-PAGE with Coomassie Blue G-250 staining.

For the purification of recombinant INS-21 and INS-23, frozen culture pellet was resuspended in 30 ml of cold PBS buffer with 1% protease inhibitor cocktail (Merck, Darmstadt, Germany) and lysed by sonication. The lysis was centrifuged at 8000 *g* and 4°C for 30 min, with the pellet being dissolved in 30 ml of PBS buffer containing 8 M urea. After another centrifugation, the supernatant was filtered through a 0.45-μm cellulose acetate membrane filter (Millipore, Billerica, MA, United States) and loaded onto Ni-NTA beads (Novagen) at 16°C and 90 rpm for 4 h. The beads were washed with 10 volumes of 6 M PBS-buffered urea containing 20 mM imidazole and eluted with 6 volumes of 6 M PBS-buffered urea containing 250 mM imidazole. The purified proteins were examined using SDS-PAGE with Coomassie Blue G-250 staining and analyzed for identity using Matrix-Assisted Laser Desorption/Ionization Time of Flight Mass Spectrometry (MALDI-TOF-MS).

### Western Blot Analysis of Native INS

Polyclonal antibodies against the recombinant INS-21 or INS-23 protein were prepared by GI Biochem Ltd. (Shanghai, China) in specific pathogen-free rabbits. Polyclonal antibodies were purified from the sera harvested from immunized rabbits using an affinity chromatographic column conjugated with purified recombinant protein. To assess the reactivity of these two antibodies, recombinant INS-21 and INS-23 were employed as the antigens in Western blot assays. Each recombinant protein (1 μg/lane) was loaded onto SDS-PAGE and transferred onto a nitrocellulose membrane. The latter was blocked with 5% non-fat milk–PBST for 2 h and incubated with anti-INS-21 (∼5.7 μg/ml) or anti-INS-23 (∼0.19 μg/ml) antibodies or pre-immune serum (1:1,000) diluted in PBS overnight at 4°C. After three washes, horseradish peroxidase (HRP)-conjugated goat anti-rabbit antibodies (Yeasen, Shanghai, China) were used as the secondary antibodies in 1-h incubation. After another three washes, the blots were treated with an enhanced chemiluminescence reagent (Thermo Fisher, Rockford, IL, United States) and analyzed with a Tanon 5200 imaging system (Tanon, Shanghai, China).

To assess the expression of the native INS proteins, the released sporozoites were washed twice with PBS buffer and resuspended in PBS containing 1% protease inhibitor cocktail (Merck, Darmstadt, Germany). They were lysed by adding protein-loading buffer and boiling for 5 min. The protein lysate (∼5 × 10^6^ sporozoites/lane) was analyzed by SDS-PAGE and transferred onto a nitrocellulose membrane, which was blocked with 5% non-fat milk-PBST for 2 h. The latter was incubated overnight at 4°C with anti-INS-21 (∼5.7 μg/ml), anti-INS-23 (∼0.19 μg/ml) antibodies, or pre-immune serum (1:1,000) dilution in PBS.

### Quantitative Analysis of INS Gene Transcription

HCT-8 cells were grown on 12-well plates until ∼90% confluence. Cells were infected with 5 × 10^5^ bleach-treated oocysts/well and washed twice with PBS at 2 hpi, and fresh 2% FBS-supplemented RPMI 1640 medium was added. RNA was extracted at specific time points using RNeasy Mini Kit (Qiagen, Hilden, Germany) and stored at -80°C until further processing. Two microliters of RNA was converted into cDNA using the GoScript Reverse Transcription System (Promega, Beijing, China). The relative expression of the cgd5_3400 and cgd7_2080 genes in intracellular parasites developed in HCT-8 cells for 0–72 h was evaluated using qPCR analysis of the cDNA. Parallel data on the expression of the *Cp*18s rRNA gene were used for data normalization. The qPCR reaction contained 0.1 mM primers, 1 μl cDNA, and 10 μl of SYBR Green PCR Mix (TOYOBO, Osaka, Japan) in a 20-μl reaction. The primers used include 5′-CATCGCTGGGAATCATCATA-3′ and 5′-CCACCTGCCAACATTAATCC-3′ for the cgd7_2080 gene (amplicon: 163 bp), 5′-GGATGAGAGTTGCAACCATGA-3′ and 5′-GCACCTAAGTCTTCAATTTGGC-3′ for the cgd5_3400 gene (amplicon: 199 bp), and 5′-CTCCACCAACTAAG AACGGCC-3′ and 5′-TAGAGATTGGAGGTTGTTCCT-3′ for the Cp18s rRNA gene (amplicon: 256 bp) (Mauzy et al., 2012). The PCR was performed on a LightCycler 480 (Roche, Basel, Switzerland), with the following cycling conditions: 95°C for 3 min and 45 cycles of 95°C for 30 s, 58°C for 30 s, 72°C for 30 s. The transcription level of the INS gene in infected HCT-8 cells was calculated by using the 2^–ΔΔ*CT*^ method (Livak and Schmittgen, 2001). The data presented were from three independent experiments performed in duplicate.

### Immunofluorescence Assay (IFA)

Sporozoites were dried onto microscopy slides and intracellular stages of *C. parvum* in HCT-8 cell cultures were grown on coverslips for 48 h. They were fixed with methanol for 15 min. After three washes with PBS, the slides were treated with 0.5% Triton-X 100 in PBS for 15 min and blocked with 5% bovine serum albumin (BSA) in PBS for 1 h. After another three washes, the slides were incubated with polyclonal antibodies against individual INS proteins (1:400 diluted in 5% BSA-PBS) for 1 h. Alexa Fluor 594-conjugated goat anti-rabbit IgG (Cell Signaling Technology, MA, United States) diluted 1:400 was used as the secondary antibodies. After 1-h incubation and three washes, the slides were counterstained with the nuclear stain 4’6-diamidino-2-phenylindole (DAPI, Roche) at room temperature for 5 min. The slides were mounted with No-Fade Mounting Medium (Booster, Wuhan, China), sealed with nail polish, and examined using differential interference contrast (DIC) and fluorescence microscopy on a BX53 microscope (Olympus, Tokyo, Japan).

### Immunoelectron Microscopy

Sporozoites were fixed at 4°C in freshly prepared mixture of 4% paraformaldehyde (Leagene, Beijing, China) and 0.1% glutaraldehyde (EMCN, Beijing, China) buffer for 60 min. Samples were washed five times with PBS buffer and embedded in 1% agarose (Macklin, Shanghai, China). They were dehydrated in a gradient ethanol series and infiltrated with LR White acrylic resin (EMCN, Beijing, China). Samples were sectioned with a Leica EM UC7 ultramicrotome and mounted on film-coated grids. The ultrathin sections of 60 nm generated were blocked with 1% BSA in PBS for 30 min and incubated at 4°C with anti-INS-21 or anti-INS-23 antibody for overnight. Following washes with 0.1% BSA in PBS, the sections were incubated at room temperature with goat anti-rabbit IgG conjugated with 10 nm colloidal gold (Sigma-Aldrich, St. Louis, MO, United States) for 60 min. They were stained with 2% uranyl acetate and Reynold’s lead citrate and viewed on a Talos L120C transmission electron microscope (Thermo Fisher Scientific, San Jose, CA, United States) equipped with Ceta-D camera (Thermo Fisher Scientific). All labeling experiments were conducted in parallel with controls omitting the primary antibody. These controls were consistently negative at the concentration of secondary antibodies used in these studies.

### Invasion Neutralization Assay

The role of individual INS in *C. parvum* invasion was assessed using a neutralization assay. Hypochlorite-treated oocysts were incubated at 37°C in culture medium containing increasing dilutions of pre-immune serum or post-immune serum for 15 min. The mixture was inoculated onto HCT-8 cells grown on coverslips in 12-well plates as described above. After 2 h of incubation, uninvaded parasites were washed off the culture with PBS and the cells were incubated for an additional 22 h in fresh culture medium. The coverslips were fixed, blocked with 5% BSA in PBS, stained with Cy3-labeled Sporo-Glo antibodies (Waterborne), and examined under a BX53 immunofluorescence microscope. Images of 50 microscope fields per coverslip were captured randomly under 200 × and analyzed using the ImageJ software^4^. The percentage of inhibition of infection was calculated by using the following formula: (1 - [No. parasites in cultures treated with post-immune serum/No. parasites in cultures treated with pre-immune serum]) × 100%. The mean percent inhibition was calculated based on data from three independent experiments. An unpaired *t*-test in GraphPad Prism 8 (GraphPad Software) was used to compare the means of two groups. For statistical analysis, two tailed *P*-values of ≤ 0.05 were considered significant.

## Results

### Expression of Recombinant INS Proteins

**FIGURE 1:**
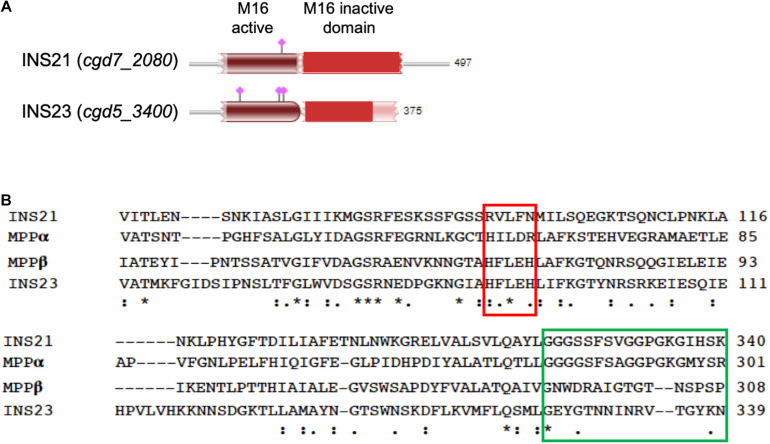
Sequence features of INS-21 and INS-23 of *Cryptosporidium parvum*. **(A)** Diagram of INS-21 (encoded by cgd7_2080) and INS-23 (encoded by cgd5_3400) of *C. parvum*. **(B)** Alignment of partial amino acid sequences of INS-21, INS-23 of *C. parvum*, and the α and β subunits of the mitochondrial processing peptidase (MPP) from *Starmerella bombicola* (yeast). Residues shown in the red box indicate the zinc-binding motif of the M16 family in MPPβ, while those in the green box represent the glycine-rich loop in MPPα. Thus, INS-23 contains the zinc-binding motifs while INS-21 contains the glycine-rich loop, indicating that both might act in concert in function. “*” indicates identical amino acid residue; “:” indicates high similarity; “.” indicates low similarity.

**FIGURE 2:**
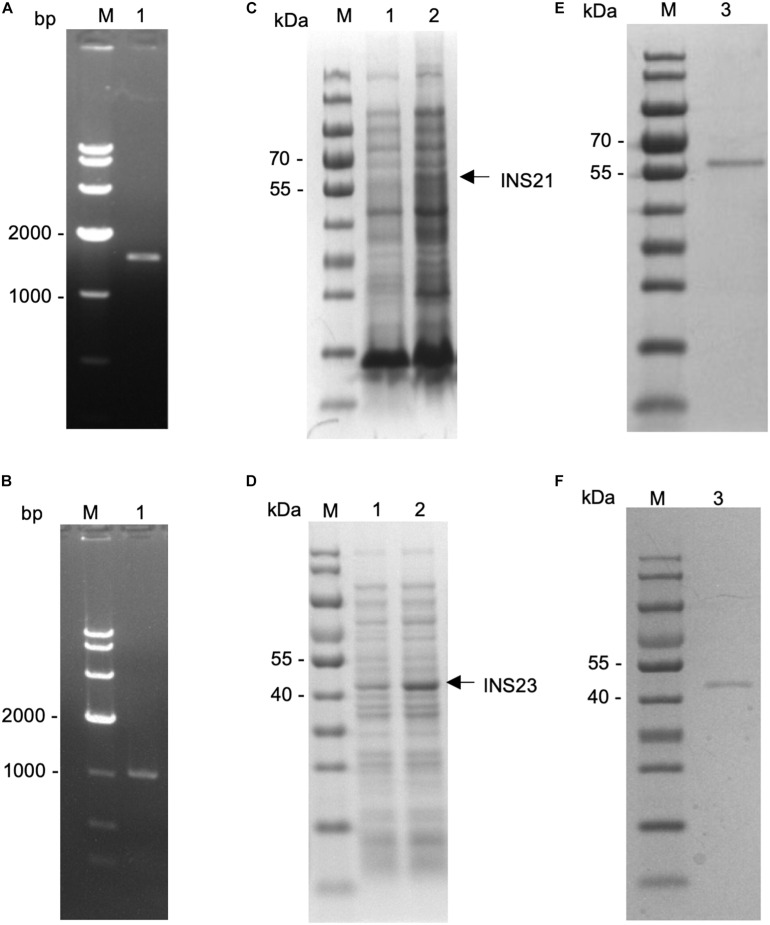
Expression of recombinant INS-21 and INS-23 in *Escherichia coli*. PCR amplification of the INS-21 gene **(A)** and INS-23 gene **(B)** of *Cryptosporidium parvum*. Lane M: 1000-bp molecular makers; Lane 1: PCR product. SDS-PAGE analysis of INS-21 protein **(C)** and INS-23 protein **(D)** expressed in *E. coli*. SDS-PAGE analysis of purified INS-21 protein **(E)** and INS-23 protein **(F)** from *E. coli*. Lane M, molecular weight markers; Lane 1, lysate from recombinant bacteria without the isopropyl β-D-thiogalactoside (IPTG) induction; Lane 2, lysate from recombinant bacteria after the IPTG induction, with the expected product being indicated by an arrow; Lane 3, purified recombinant proteins from the *E. coli* lysate using Ni-NTA affinity chromatography.

The predicted INS-23 encoded by the cgd5_3400 gene has two domains: one active domain containing the zinc-binding motif “HFLEH” and one inactive domain without it. In contrast, INS-21 encoded by the cgd7_2080 gene has two inactive domains and a glycine-rich loop at the C-terminus (Figure 1). In order to obtain the recombinant proteins of INS-21 and INS-23, the cgd5_3400 and cgd7_2080 genes were amplified by PCR (Figures 2A,B) from genomic DNA of *C. parvum* and cloned into the pET-28a vector. Recombinant INS proteins were expressed in *E. coli* (Figures 2C,D) and purified using the His-tag incorporated (Figures 2E,F). In SDS-PAGE analysis, the expected bands of INS-21 and INS-23 were seen at ∼60 kDa and ∼45 kDa in agreement with the predicted sizes (Supplementary Table 1), respectively. The identity of the products was confirmed using the MALDI-TOF-MS analysis, yielding peptide sequences of each INS (Supplementary Table 1 and Supplementary Figure 1). The polyclonal antibodies against recombinant INS-21 or recombinant INS-23 were generated by immunizing rabbits.

### Reactivity of Anti-INS Antibodies to Native INS Proteins

In order to evaluate the reactivity of these antibodies to native INS proteins, we firstly tested the reactivity of anti-INS antibodies to recombinant INS-21 and INS-23 using Western blot. The anti-INS-21 antibodies reacted strongly with recombinant INS-21 protein and lightly with recombinant INS-23 protein. In contrast, the anti-INS-23 antibodies only recognized recombinant INS-23 protein, with no cross-reactivity with recombinant INS-21 protein (Supplementary Figure 2).

**FIGURE 3:**
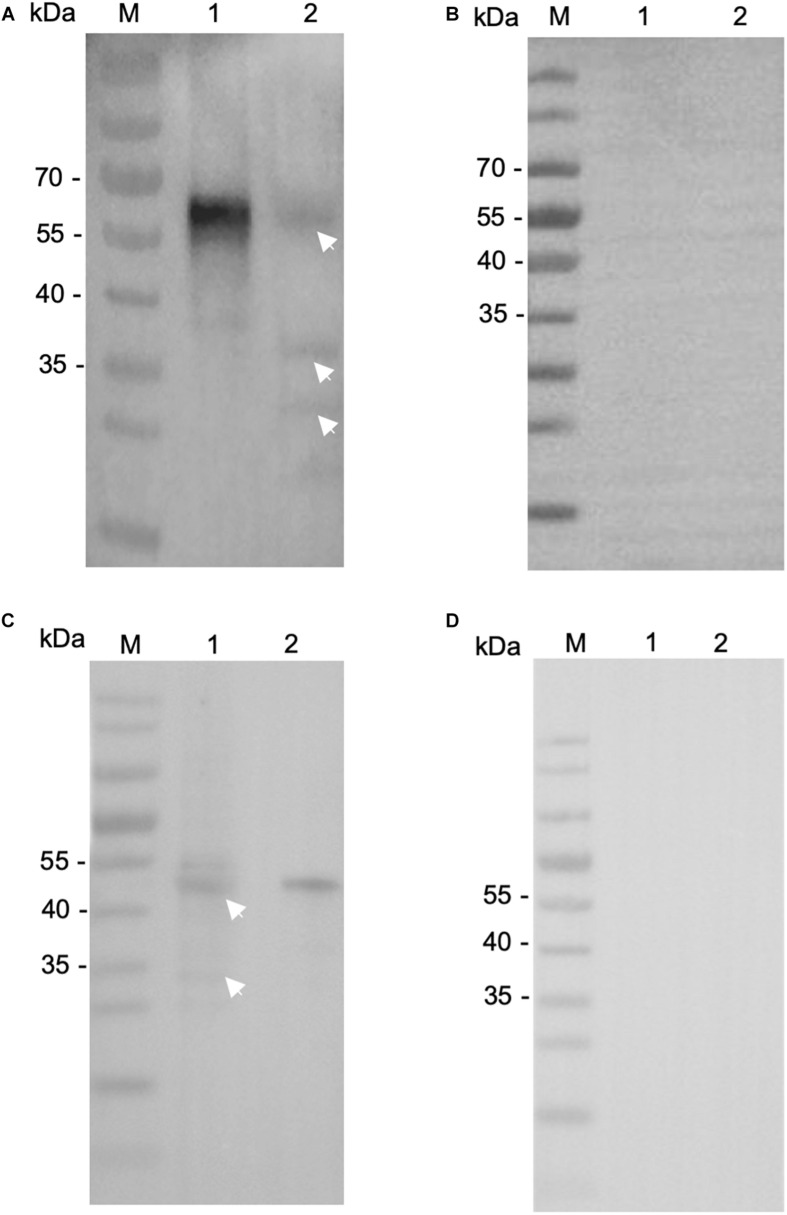
Expression of native INS-21 and INS-23 proteins in sporozoites of *Cryptosporidium parvum*. Western blot analysis of native INS-21 protein using antibodies against INS-21 **(A)** and pre-immune serum **(B)**. Lane M, molecular weight markers; Lane 1, purified recombinant INS-21 protein; Lane 2, native proteins from sporozoites. Furthermore, the expression of native INS-23 protein was analyzed by Western blot using antibodies against INS-23 **(C)** and pre-immune serum **(D)**. Lane M, molecular weight markers; Lane 1, native proteins from sporozoites; Lane 2, purified recombinant INS-23 protein. Arrows indicate native proteins from sporozoites reacting with polyclonal antibodies.

We then evaluated the reactivity of these antibodies to native INS proteins. In Western blot, the anti-INS antibodies recognized their corresponding INS proteins in crude extracts of sporozoites, while pre-immune serum did not (Figures 3B,D). The anti-INS-21 antibodies reacted with a protein of ∼60 kDa and two proteins smaller than 40 kDa in sporozoite lysate (Figure 3A), while the anti-INS-23 antibodies reacted with a protein of ∼45 kDa (Figure 3C) and a few proteins smaller than 35 kDa, suggesting that INS-21 and INS-23 might be expressed in sporozoites, and there could be proteolytic processing of these INS.

### Transcription of INS-21 and INS-23 Genes in Developmental Stages of *C. parvum*

**FIGURE 4:**
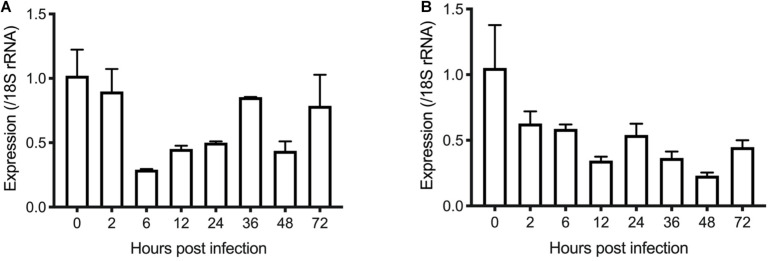
Transcription levels of the *INS-21* **(A)** and *INS-23* genes **(B)** in developmental stages of *C. parvum*. The relative expression of the INS gene at various *C. parvum* cultivation time was determined by reverse transcription-qPCR, with data being normalized with data from the expression of the *Cp*18S rRNA gene.

To examine the transcription of the INS-21 and INS-23 genes during the intracellular development of *C. parvum in vitro*, we infected HCT-8 cells with oocysts and tested transcription of the INS genes at different time points using reverse transcription-quantitative PCR (RT-qPCR). Both the cgd5_3400 and cgd7_2080 had the highest transcription at 0 h post infection (hpi). At this time point, all parasites were sporozoites in the culture, indicating that the expression of the INS-21 and INS-23 gene was at the highest level in this developmental stage. The cgd7_2080 gene, *INS-21*, kept a high transcription at 2 hpi and maintained some expression until 72 hpi (Figure 4A). The transcription of the cgd5_3400 gene, *INS-23*, decreased at 2 hpi compared with 0 hpi, but was maintained at relatively high levels until 72 hpi (Figure 4B), indicating that both INS-21 and INS-23 genes are probably transcribed in both asexual parasites and sexual parasites (Tandel et al., 2019; Funkhouser-Jones et al., 2020).

### Localization of INS-21 and INS-23 in Sporozoites and Intracellular Stages

**FIGURE 5:**
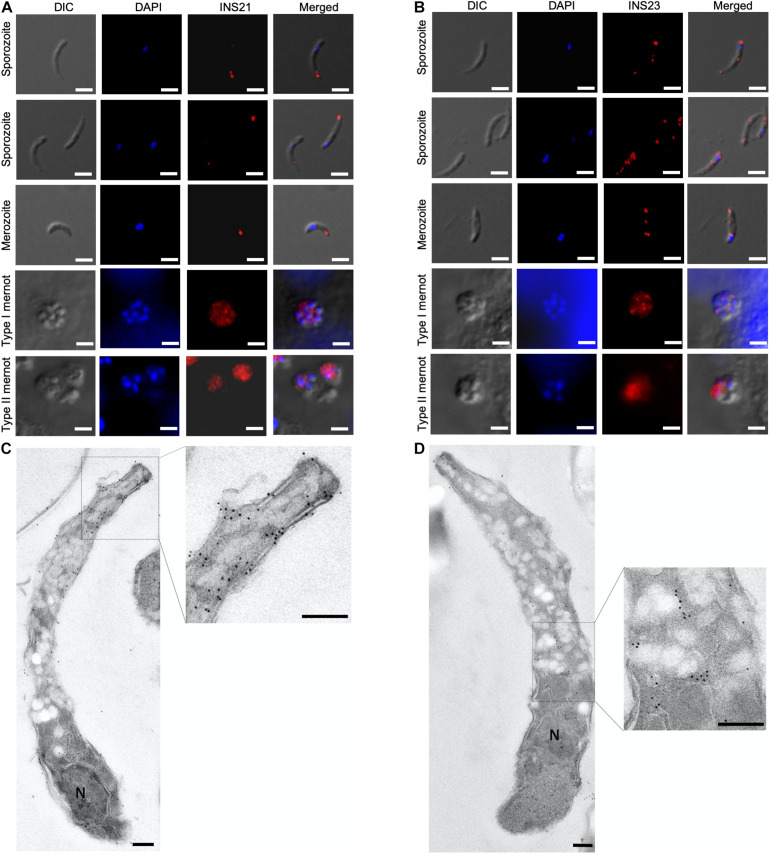
Expression of INS proteins in *C. parvum* life cycle stages indicated by immunofluorescence and transmission electron microscopy. Expression of INS-21 **(A)** and INS-23 **(B)** in *C. parvum* sporozoites and intracellular developmental stages in HCT-8 cell cultures, such as merozoites, type I meronts, and type II meronts, was examined using immunofluorescence microscopy. The images were taken under differential interference contrast (DIC), with the nuclei being counter-stained with 4’,6-diamidino-2-phenylindole (DAPI), parasites stained by immunofluorescence with anti-INS antibodies, and superimposition of the three images (Merged). Scale bars = 2 μm. In addition, the expression of the two INS in sporozoites was examined using transmission electron microscopy with anti-INS-21 antibodies **(C)** or anti-INS-23 antibodies **(D)** followed by the use of 18-nm colloidal gold-labeled goat anti-rabbit IgG. N, nucleus; AC, apical complex. Scale bars = 200 nm.

To visualize the stage-specific location of INS-21 and INS-23 expression, we performed immunofluorescence microscopy using antibodies against each INS. Anti-INS-21 antibodies had strong reaction with the apical region of sporozoites, while anti-INS-23 antibodies reacted with multiple areas of sporozoites with a dotted pattern. In the intracellular stage, anti-INS-21 and anti-INS-23 antibodies reacted with merozoites in patterns similar to those in sporozoites. Both antibodies also reacted with merozoites within type I and type II meronts (Figures 5A,B), consistent with the transcription level of the gene encoding each protein.

The organelles expressing INS-21 and INS-23 in sporozoites were assessed by immunoelectron microscopy. INS-21 was located in the anterior of sporozoites, probably the micronemes (Figure 5C), while INS-23 was located in almost the entire sporozoites in a dotted pattern, probably in dense granules (Figure 5D). Because of the lack of antibodies to known markers of most organelles in *Cryptosporidium* spp., it was impossible to use the co-localization approach to confirm the identification of organelles expressing INS-21 and INS-23.

### Inhibition of *C. parvum* Invasion by Anti-INS Antibodies

**FIGURE 6:**
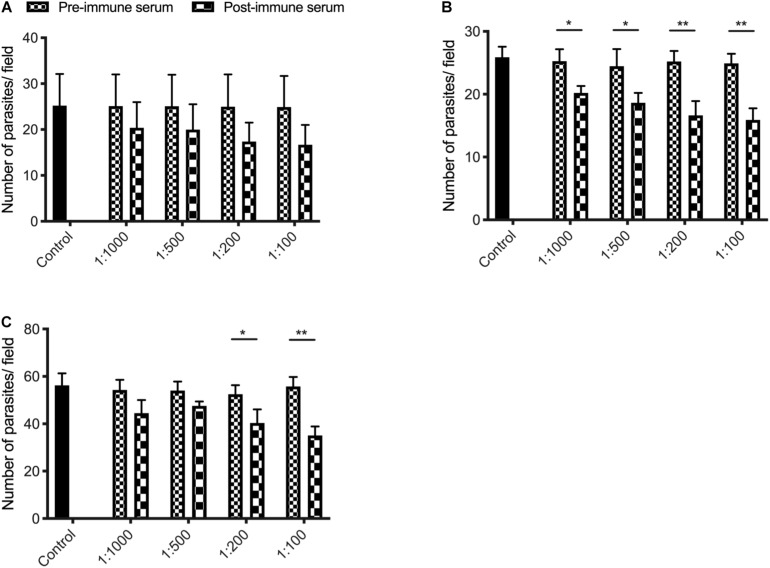
Inhibition efficiency of immune sera against INS-21 **(A)**, INS-23 **(B)**, and the combination of the two **(C)** on *C. parvum* invasion. Inhibition efficiency of *C. parvum* invasion by immune sera against INS was measured in HCT-8 cell culture. Data presented are mean ± SD from three independent experiments for both the expression and neutralization studies. Statistical analysis was performed using unpaired *t*-test for pairwise comparisons (*P < 0.05; **P < 0.01).

We assessed the effect of anti-INS antibodies on *C. parvum* invasion by incubating bleached oocysts with anti-INS antibodies and keeping the antibodies in the media during the sporozoite infection of HCT-8 cells. Compared with the control culture, the cultures treated with antiserum against INS-23 or treated with antiserum against both INS-21 and INS-23 had a modest but significant reduction in parasite load. When the cultures were treated with antiserum INS-21, there was no significant reduction in parasite load. The inhibitory effect of antiserum against INS-21 was 18.8% [*t*_(2)_ = 0.9207, *P* = 0.4093] at 1:1000 dilution, 20.4% [*t*_(2)_ = 1.004, *P* = 0.3722] at 1:500 dilution, 30.5% [*t*_(2)_ = 1.616, *P* = 0.1813] at 1:200 dilution, and 33.0% [*t*_(2)_ = 1.768, *P* = 0.1519] at 1:100 dilution (Figure 6A). In contrast, the inhibitory effect of antiserum against INS-23 was 20.2% [*t*_(2)_ = 4.003, *P* = 0.0161] at 1:1,000 dilution, 24.1% [*t*_(2)_ = 3.191, *P* = 0.0332] at 1:500 dilution, 34.1% [*t*_(2)_ = 5.255, *P* = 0.0063] at 1:200 dilution, and 36.1% [*t*_(2)_ = 6.488, *P* = 0.0029] at 1:100 dilution (Figure 6B).

The inhibitory effect of *C. parvum* cultures with antisera against both INS-21 and INS-23 was 18.1% [*t*_(2)_ = 2.438, *P* = 0.0714] at 1:1,000 dilution, 11.9% [*t*_(2)_ = 2.615, *P* = 0.0591] at 1:500 dilution, 23.1% [*t*_(2)_ = 3.069, *P* = 0.0373] at 1:200 dilution, and 37.1% [*t*_(2)_ = 6.534, *P* = 0.0028] at 1:100 dilution (Figure 6C).

## Discussion

Data from the present study suggest that INS-21 and INS-23 may have slightly different roles in the invasion and development of *C. parvum*. Both INS-21 and INS-23 have high expression at the time of host cell invasion by sporozoites (Borowski et al., 2010). Despite some cross-reactivity of anti-INS-21 antibodies to recombinant INS-23, immunofluorescence and immunoelectron microscopy studies with these antibodies suggest that INS-21 is expressed in micronemes of sporozoites and possibly also merozoites, while INS-23 is likely expressed in dense granules of these invasive stage. However, the antibodies against the INS-21 or INS-23 only had a modest inhibition of parasite invasion in host cells, probably because antibody neutralization is not ideal in assessing the biological functions of proteases, and most apicomplexans use multiple strategies for invasion (Walker et al., 2014).

Data accumulated thus far have shown different expression profiles of INS in *C. parvum*. Several INS proteins of *C. parvum* have been studied thus far, including INS-5, INS-15, and INS-20-19. INS-15 is a classic M16A family protein with four conserved domains (Xu et al., 2019), INS-20-19 is a species-specific protein that is absent in *C. hominis* (Zhang et al., 2019). Both INS-15 and INS-20-19 have another paralog in *C. parvum*. In contrast, INS-5 contains only one inactive M16 domain (Ni et al., 2020). Although the INS-15, INS-20-19, INS-21, and INS-23 genes all have high expression at 0–2 h of the *in vitro* infection, the patterns of the protein expression in *C. parvum* are different among them. INS-20-19 and INS-21 are expressed in the apical region of sporozoites, INS-15 proteins are expressed in the mid-anterior area of sporozoites, while INS-23 has a spotted pattern over the entire sporozoites (Xu et al., 2019; Zhang et al., 2019). In immunoelectron microscopy, INS-21 appears to be expressed in the micronemes, while INS-23 probably in dense granules. In contrast, INS-5 is an another M16B protein containing only one inactive domain and with the highest gene expressed at 36–48 hpi (Ni et al., 2020). In addition, antibodies against these INS proteins have been shown to inhibit parasite invasion *in vitro*, suggesting that they might be expressed in different organelles of the parasite and therefore exert different functions in the invasion and development of *C. parvum*.

INS-21 and INS-23 might play their functions in concert in *C. parvum*. The M16B proteases in eukaryotes are approximately 500 amino acids in length, with two subunits encoded by two separate genes. The best-studied M16B protease is the mitochondrial processing peptidase (MPP) of yeasts, which consists of an α subunit and a β subunit, with the β subunit containing an “HXXEH” motif (Taylor et al., 2001). In this study, we have focused on two of the three M16B proteases in *C. parvum*, INS-21 and INS-23. In the sequence alignment of them together with MPP from yeasts (Figure 1B), INS-23 protein containing 375 amino acids is more similar to the β subunit. Like in the β subunit, INS-23 protein also has a zinc-binding motif “HFLEH” in the M16 active domain. In contrast, INS-21 protein containing 497 amino acids is more similar to the α subunit, with a glycine-rich loop in the C-terminus that protrudes into the active site, and is thus essential for catalysis (Nagao et al., 2000).

The preliminary evidence generated in the study suggests that INS proteins may play some roles in *Cryptosporidium* life cycle, as they have high expression levels in the early development stage of *C. parvum*. More biologic and immunohistological studies, however, are needed to better understand the mechanism of INS-21 and INS-23 proteins during invasion and development of *C. parvum*. Based on the nature of M16B enzymes, the interaction of these two proteins should be elucidated using co-immunoprecipitation or crystal structure analysis. These advanced studies will likely lead to better understanding of the functions of INS proteases in *Cryptosporidium* life cycle.

## Data Availability Statement

All datasets generated for this study are included in the article and/or the Supplementary Material.

## Author Contributions

YF and LX conceived and designed the experiments. RX, CL, and FY performed the experiments. YG, NL, and QZ provided technical assistance. RX, YF, and LX analyzed the data and wrote the manuscript. All authors contributed to the article and approved the submitted version.

## Conflict of Interest

The authors declare that the research was conducted in the absence of any commercial or financial relationships that could be construed as a potential conflict of interest.

## Footnotes

1 http://cryptodb.org

2 http://pfam.xfam.org

3 https://www.ebi.ac.uk/Tools/msa/clustalo/

4 https://imagej.nih.gov/ij/
