# Supplementary material for: Preliminary Characterization of Two Small Insulinase-Like Proteases in Cryptosporidium parvum
Source: Front Microbiol. 2021 May 21;12:651512. doi: 10.3389/fmicb.2021.651512 (PMC8175991; doi:10.3389/fmicb.2021.651512)

Table S1: Proteins identified in SDS-PAGE bands by MALDI-TOF-MS analysis

| Sample | Protein name | Accession No. | Protein MW (kDa) | Protein PI | Protein Score | Protein Score C. I. % |
| --- | --- | --- | --- | --- | --- | --- |
| INS21 | Mitochondrial processing peptidase, insulinase like metalloprotease | XP_628385.1 | 56.21 | 8.67 | 106 | 100 |
| INS23 | Mitochondrial processing peptidase beta subunit | XP_001388286.1 | 42.96 | 8.82 | 340 | 100 |

Figure S1: Peptide coverage of INS21 and INS23 by MALDI-TOF-MS analysis

(a) Peptides coverage of INS21 by MALDI-TOF-MS analysis. Purified recombinant INS21 was examined using the SDS-PAGE, with the gel being stained with Coomassie Blue G-250. The expected band was cut and analyzed by MALDI-TOF-MS. Peptides with the underlines were identified by MALDI-TOF-MS.

(b) Peptide coverage of INS23 by MALDI-TOF-MS analysis. Purified recombinant INS23 was examined using the SDS-PAGE, with the gel being stained with Coomassie Blue G-250. The expected band was cut and analyzed by MALDI-TOF-MS. Peptides with the underlines were identified by MALDI-TOF-MS.


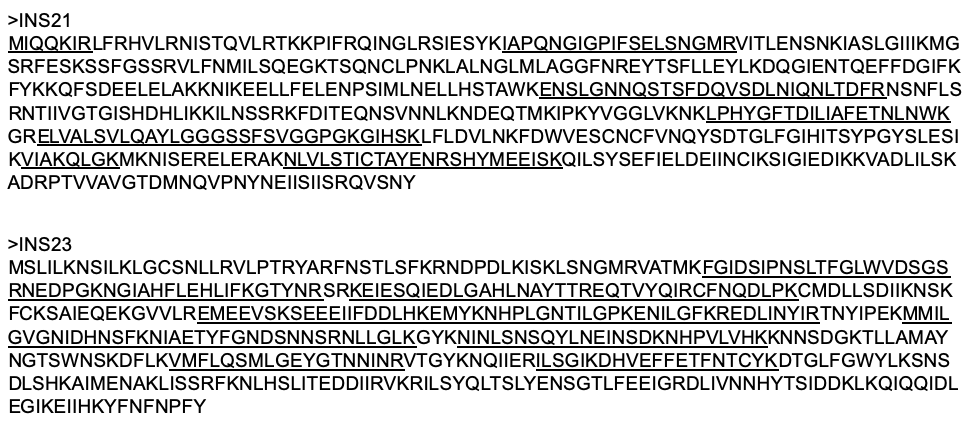
a

b

Figure S2: Cross-reactivity of polyclonal antibodies against INS-21 and INS-23. Cross-reactivity of anti-INS-21 antibodies (a) and pre-immune serum (b). Cross-reactivity of anti-INS-23 antibodies (c) and pre-immune serum (d). Lane M: molecular weight markers; Lane 1: purified recombinant INS-21 protein; Lane 2: purified recombinant INS-23 protein.


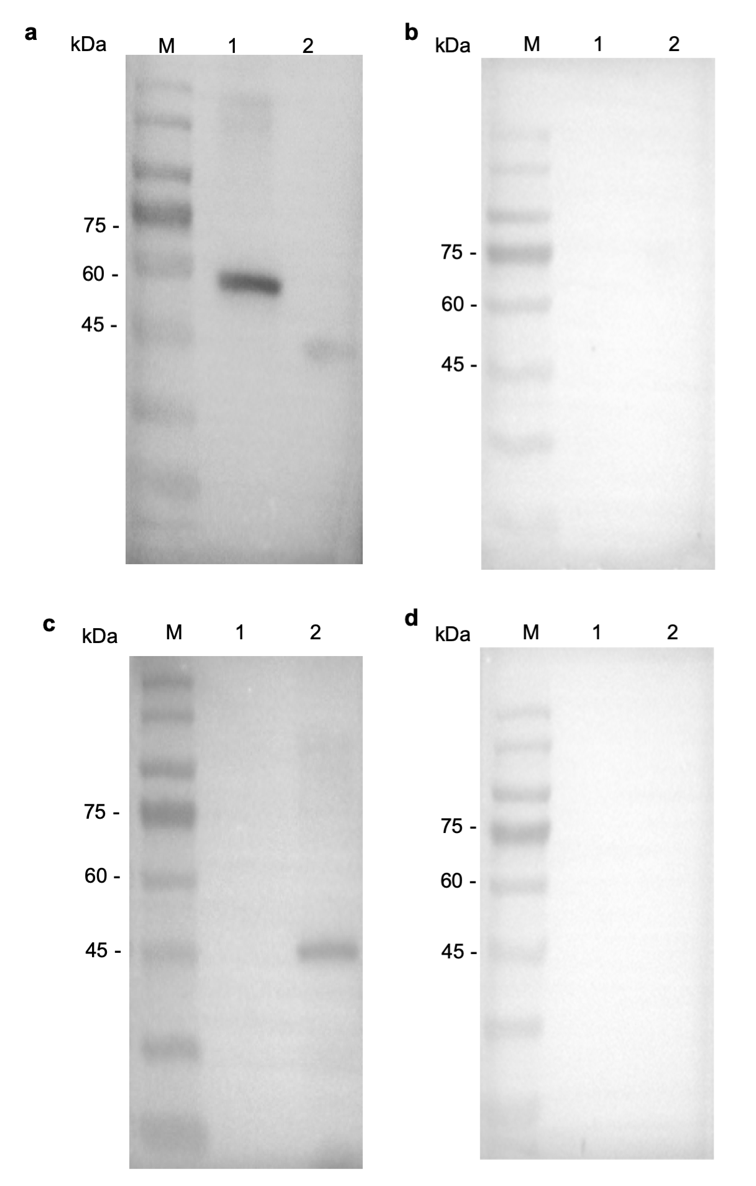

Supplement: Supplementary file 1 [file Data_Sheet_1.docx]
